# AI‐derived prognostic biomarkers from melanoma whole slide image segmentation: an initial discovery and assessment

**DOI:** 10.1002/2056-4538.70075

**Published:** 2026-03-04

**Authors:** Emily L Clarke, Derek Magee, Julia Newton‐Bishop, Gerald Saldanha, William Merchant, Marlous Hall, Robert Insall, Nigel G Maher, Richard A Scolyer, Grace Farnworth, Anisah Ali, Mark Bamford, Eva Sticova, Petr Kujal, Sally O'Shea, Darren Treanor

**Affiliations:** ^1^ Department of Histopathology Leeds Teaching Hospitals NHS Trust Leeds UK; ^2^ Division of Pathology and Data Analytics University of Leeds Leeds UK; ^3^ School of Computing University of Leeds Leeds UK; ^4^ HeteroGenius Limited Leeds UK; ^5^ Leeds Institute for Medical Research University of Leeds Leeds UK; ^6^ Leicester Cancer Research Centre University of Leicester Leicester UK; ^7^ Department of Histopathology University Hospitals of Leicester NHS Trust Leicester UK; ^8^ Department of Clinical and Population Sciences, Leeds Institute of Cardiovascular and Metabolic Medicine University of Leeds Leeds UK; ^9^ Department of Cell and Developmental Biology University College London London UK; ^10^ Melanoma Institute Australia The University of Sydney Sydney New South Wales Australia; ^11^ Faculty of Medicine and Health The University of Sydney Sydney New South Wales Australia; ^12^ Tissue Pathology and Diagnostic Oncology Royal Prince Alfred Hospital and NSW Health Pathology Sydney New South Wales Australia; ^13^ Charles Perkins Centre The University of Sydney Sydney New South Wales Australia; ^14^ Department of Pathology, Third Faculty of Medicine Charles University and Teaching Hospital Kralovske Vinohrady Prague Czech Republic; ^15^ Dermatology Department South Infirmary Victoria University Hospital Cork Ireland; ^16^ School of Medicine University College Cork Cork Ireland; ^17^ Department of Clinical Pathology, and Department of Clinical and Experimental Medicine Linköping University Linköping Sweden; ^18^ Center for Medical Image Science and Visualization (CMIV) Linköping University Linköping Sweden

**Keywords:** melanoma, histology, digital pathology, artificial intelligence, convolutional neural networks, machine learning, biomarkers

## Abstract

The current melanoma staging system predicts 74% of the variance in survival, with prognostic biomarkers subject to high levels of inter‐observer variation. This work assesses whether a previously developed convolutional neural network (CNN) for invasive melanoma segmentation in whole slide images (WSIs) may reveal new insights into melanoma morphology and patient prognosis. This paper uses Cox proportional multivariate regression analyses to evaluate the ability of the CNN outputs to predict patient survival across 745 WSIs from 5 data sources. Five objective histomorphological parameters of tumour size and shape that are independently associated with overall and melanoma‐specific survival were created from the CNN: tumour area*(log)* (HR 1.48 CI 1.30–1.68, *p* < 0.001), tumour perimeter*(log)* (HR 1.86 CI 1.48–2.32, *p* < 0.001), major axis length*(log)* (HR 1.88 CI 1.42–2.48, *p* < 0.001), Nodularity Index*(log)* (HR 1.77 CI 1.28–2.43, *p* < 0.001) and digital Breslow thickness*(log)* (HR 2.04, CI 1.63–2.54, *p* < 0.001). These results indicate that melanoma segmentation of the entire lesion within a WSI may be used to predict patient outcome. Moreover, this technology can be used to make new morphological discoveries to provide information not currently contained within our staging system (*e.g.* Nodularity Index), as well as provide objectivity and automation of current biomarkers (*e.g.* digital Breslow thickness). Further work is required to validate this initial discovery and evaluation.

## Introduction

As summarized in a previous study [1], convolutional neural networks (CNNs) have been used increasingly over the past decade in health research to improve workflow by automating tasks or providing new insights into disease [2]. Of late, this technology has spread to histopathology, facilitated through the introduction of whole slide imaging, where it is hoped that CNNs may aid in screening/diagnostic triage or with the identification of new prognostic or predictive biomarkers. However, progression in the field of melanoma has fallen behind other areas (*e.g*. breast and prostate), largely due to the complexity and variety of melanoma histomorphologies and the relative scarcity of specimens with long‐term follow‐up data.

The morphology of melanoma has been thought to be of greater significance than in other histopathological fields [1]. This is exemplified by a morphological parameter of tumour size, Breslow thickness, remaining the most important prognostic biomarker in this disease more than 50 years after its discovery [3], despite significant inter‐observer variation [4]. More recently, the work of Saldanha *et al* into the width of invasion [5], Breslow Density [6] and Calculated Tumour Area [7], as assessed subjectively by histopathologists, has highlighted the possibility for more detailed morphological assessment in melanoma leading to improvements in prognostication. As is aptly highlighted by McCalmont in JAMA Dermatology, the use of technology to objectify these assessments could reveal new features to better stratify patients [8].

To date, many papers have focused on a multimodal approach utilizing existing clinicopathological information and/or gene expression profiles for survival prediction [9] or segmentation of melanoma to help with diagnostic support or triage. We are unaware of any published articles concerned with the use of a neural network to segment the whole lesion to provide a novel histopathological assessment of the tumour in melanoma for prognostication. Moreover, there has been no investigation into the effect of tumour shape independent of size on survival. We hypothesize that our CNN, which can segment conventional invasive melanoma accurately [1], may be able to be used to derive objective prognostic biomarkers based on the tumour size and shape. Therefore, the objective of this work is to create and evaluate a prognostic biomarker(s) based on the tumour morphology using the outputs of the CNN outlined in our previous work [1].

## Materials and methods

This paper utilizes a custom‐designed, 2‐class, fully convolutional neural network to segment all pixels in the WSI into ‘invasive melanoma’ and ‘not invasive melanoma’, trained using manual annotation. Details regarding the development and evaluation are detailed in a previously published work [1].

### Deriving objective morphological tumour parameters

Using Medical Image Manager (HeteroGenius Limited, Leeds, UK) the area (mm^2^), perimeter (mm), major axis length (mm), minor axis length (mm) and Nodularity Index (NI) of the largest piece of segmented invasive melanoma were calculated for each WSI. Area and perimeter were generated directly from the segmentation outputs of the CNN. Major and minor axis lengths were produced using the rotating callipers algorithm [10] to create a bounding box encapsulating the CNN segmented tumour. The major axis length is the largest length of the bounding box, and the minor axis length is the smallest. The identification of only the largest piece of tumour eradicates the issue of serially sliced specimens with multiple tissue sections containing tumour embedded in one block.

It is highly likely that larger tumours (*i.e*. those with a higher area, major and minor axis lengths) will have a worse prognosis and, therefore, metrics concerned solely with tumour size are likely to be prognostic of survival, with higher values being associated with a worse prognosis. A graphical presentation of the major and minor axis lengths can be seen in supplementary material, Figure S1.

**Figure 1:**
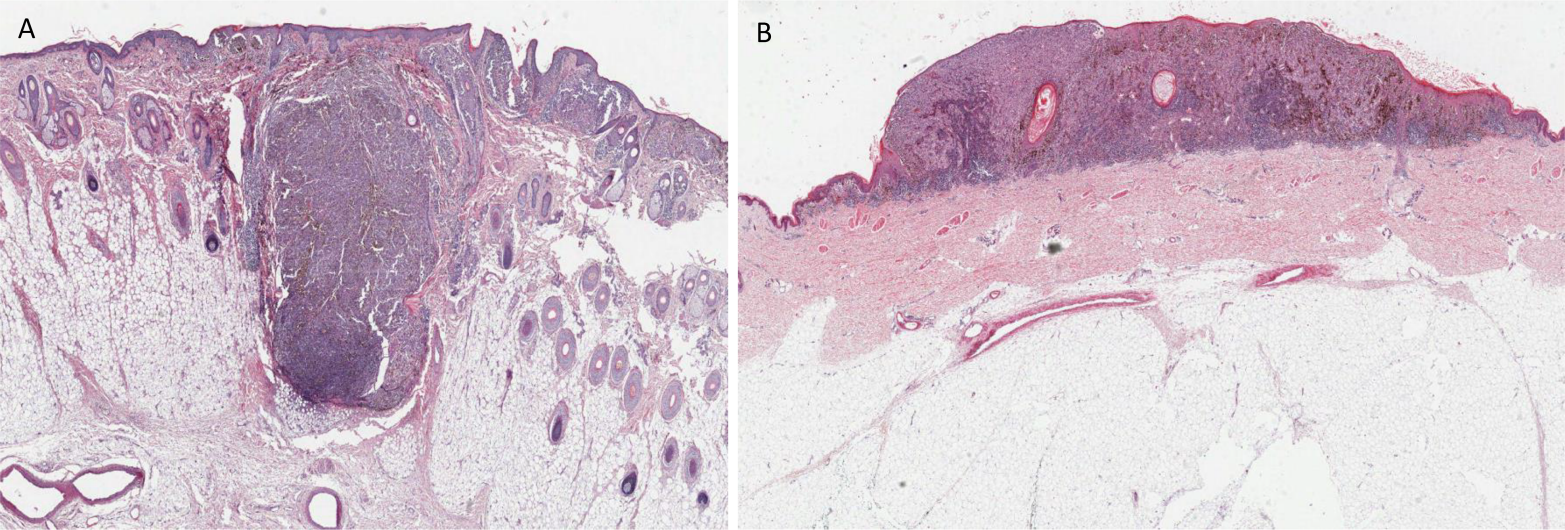
An example of the differences in melanoma shape. Part figure (A) is a melanoma with a higher Nodularity Index than (B). Major subtype is likely to be highly correlated with Nodularity Index.

The NI (Figure 1) is a ratio between the major and minor lengths and is therefore a metric of tumour shape independent of the tumour size. It is a measure of tumour depth per unit width. This may be thought of as the ‘roundness’ or ‘nodularity’ of a tumour and is likely to be closely correlated with major subtype (superficial spreading melanoma *vs* nodular melanoma). It is hypothesized that the more nodular the tumour (or greater depth per unit width), the higher the NI. Although it is well documented that nodular melanomas confer a worse prognosis, this is not known to be the case when standardized for size (*i.e*. nodular melanomas often have a larger Breslow thickness as they are commonly diagnosed at a later stage). However, isolating a ‘shape‐only’ metric is not possible through standard histopathological review.

### Survival analysis

Using the morphological outputs of the CNN as described above, a survival analysis was conducted using 745 WSIs (745 cases from 745 patients) from across 5 data sources. These cases had not been included in training or validation and included non‐fragmented full‐face melanomas, including all 4 major subtypes, as shown in Table 1. These 745 cases included 479 cases from the Leeds Melanoma Cohort (LMC), 91 cases from the Vitamin D and Immunity in Melanoma Dataset (VDI), 75 cases from the Melanoma Institute Australia (MIA), 83 cases from the University of Leicester (UHL) and 17 cases from the Charles University (CU) and Teaching Hospital Kralovske Vinohrady, Prague.

**Table 1:** Background characteristics of the 745 cases included in the melanoma detection CNN survival analysis

Background characteristic	Number (%)
Age/years	
<50 years	181 (24)
≥50 years	564 (76)
Unknown	0 (0)
Sex	
Male	376 (50)
Female	369 (50)
Unknown	0 (0)
Breslow thickness/mm	
≤1.0 mm	82 (11)
>1.0–2.0 mm	294 (40)
>2.0–4.0 mm	255 (34)
>4.0 mm	112 (15)
Unknown	2 (0)
AJCC Clinical Stage Group at diagnosis	
IA	21 (3)
IB	198 (27)
IIA	126 (17)
IIB	91 (12)
IIC	28 (4)
IIIA	32 (4)
IIIB	54 (7)
IIIC	30 (4)
IVA	0 (0)
IVB	7 (1)
Not stated	158 (21)
Subtype	
Superficial spreading	442 (59)
Nodular	224 (30)
Lentigo maligna	16 (2)
Acral	16 (2)
Unknown	47 (6)
Site	
Central	374 (50)
Peripheral	368 (50)
Unknown	3 (0)
Regression	
Present	123 (17)
Absent	444 (60)
Unknown	178 (24)
Ulceration	
Present	235 (32)
Absent	469 (63)
Unknown	41 (6)
Microsatellitosis	
Present	41 (5)
Absent	700 (94)
Unknown	4 (1)
Vascular invasion	
Present	62 (8)
Absent	557 (75)
Unknown	126 (17)
Perineural invasion	
Present	16 (2)
Absent	385 (52)
Unknown	344 (46)
*BRAF* mutation status	
Wildtype	68 (10)
Mutant	40 (5)
Unknown	637 (86)

*BRAF* mutation status was available for a subset of cases within the LMC, MIA and Prague datasets. *BRAF* mutations of all types were coded as ‘mutant’. Twenty‐eight (70%) cases were *BRAF* V600E mutations, 11 (28%) cases were V600 non‐E mutations, and 1 case was a non V600 mutation.

The test set included all 4 main subtypes (superficial spreading, nodular, lentigo maligna, and acral melanomas) and included pigmented and non‐pigmented tumours. However, as they are less common tumours, lentigo maligna melanomas and acral melanomas formed the minority of the test set, accounting for just 4%. Tumours that formed one continuous nodule, as well as those formed by numerous nests, were also included. However, melanomas of ‘special type’ were excluded, *e.g*. spitzoid, spindle, desmoplastic, *etc*.

To estimate the association between the morphological parameters and survival, we first generated unadjusted Kaplan–Meier estimates and tested differences between quartiles of each parameter using the log‐rank test. Subsequently, Cox proportional hazard models were used to evaluate the adjusted association between the CNN‐derived parameter of tumour shape and overall survival (OS), as well as melanoma‐specific survival (MSS). A directed acyclic graph (DAG) informed our choice of confounders and excluded mediators. The models were thus adjusted for age, sex and site, since there is empirical evidence that these variables affect both patient survival and tumour size/shape. Site was categorized into central *versus* peripheral, with head and trunk coded as central and limbs as peripheral. AJCC stage, Breslow thickness, ulceration, regression, subtype, perineural invasion, vascular invasion and microsatellitosis were not included in the model, since these parameters are likely to be on the same causal pathway as the CNN‐derived tumour shape parameters. Spearman's correlation coefficients confirmed that age, sex and site were not co‐linear. Bootstrapping by lossless non‐parametric resampling with replacement with 1000 replications was performed to improve the precision of the model estimates. To compare the predictive power of the new morphological parameters and Breslow thickness, Harrell's *C* statistic was used (concordance index). All morphological parameters were included in the models as continuous variables with log transformation to ensure stability of the hazard ratio given these were consistently rightly skewed. The proportional hazards assumption was met through examination of plots of Schoenfield residuals and goodness‐of‐fit tests. Across the full dataset, the median follow‐up period was 5.7 years (maximum 33.7 years). The proportion of patients who died during the follow‐up period was 30%. Subgroup analyses were conducted to investigate morphological outputs with known important morphological features in melanoma using Wilcoxon rank‐sum tests and logistic regression. To reduce the risk of type 2 error, the family‐wise error rate was revised down to *p* ≤ 0.001 for all analyses. All statistical analysis was conducted using Stata MP, Version 18 (Brownsville, TX).

This paper, in combination with our previously published work [1] outlining the development and evaluation of the model, has been prepared with reference to the Tripod‐AI statement [11].

This work was conducted under the national system for ethical approval by the Cambridge East Research Ethics Committee (19/EE/0385).

## Results

### Case characteristics

The background characteristics of the 745 cases from across the 5 test sets used for the survival analysis can be seen in Table 1.

The average values and distributions for each of the tumour parameters for the largest piece of tumour are shown in supplementary material, Table S1.

The minor axis length was highly positively correlated with standard Breslow Thickness (Spearman's rho = 0.89). The minor axis length is therefore termed the ‘digital Breslow thickness’ (dBT) going forwards.

### Grouped morphological parameters

**Figure 2:**
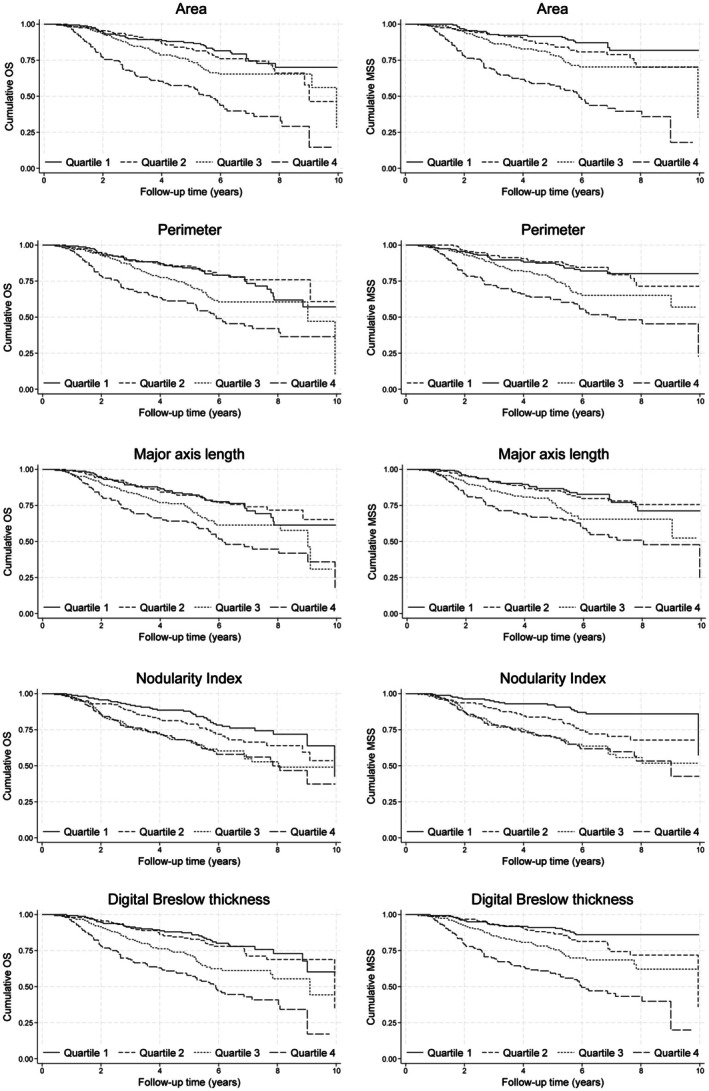
Kaplan–Meier plots for each of the parameters. Plots showing overall survival can be seen on the left, and melanoma‐specific survival on the right. There were significant differences between quartiles for overall survival and melanoma‐specific survival for all parameters. Cases with a higher area, perimeter, major axis length, digital Breslow thickness, and higher Nodularity Index were associated with poorer survival.

When each of the parameters was split into quartiles (equal number of cases in each group), there were highly significant differences in terms of overall survival (OS) and melanoma‐specific survival (MSS) for tumour area (*p* < 0.001 and *p* < 0.001, respectively) and perimeter (*p* < 0.001 and *p* < 0.001, respectively). Larger areas and perimeters (higher quartiles) were associated with worse survival. Similarly, both the major axis lengths and dBT were similarly significant in terms of survival (*p* < 0.001 and *p* < 0.001, respectively), with the higher quartiles (larger lengths) having worse survival. NI bore a similarly significant relationship with OS and MSS (*p* < 0.001 and *p* < 0.001, respectively), whereby the higher the NI (more rounded with greater depth per unit width), the worse the survival. Kaplan–Meier plots for each of the parameters can be seen in Figure 2.

### Cox regression modelling

**Figure 3:**
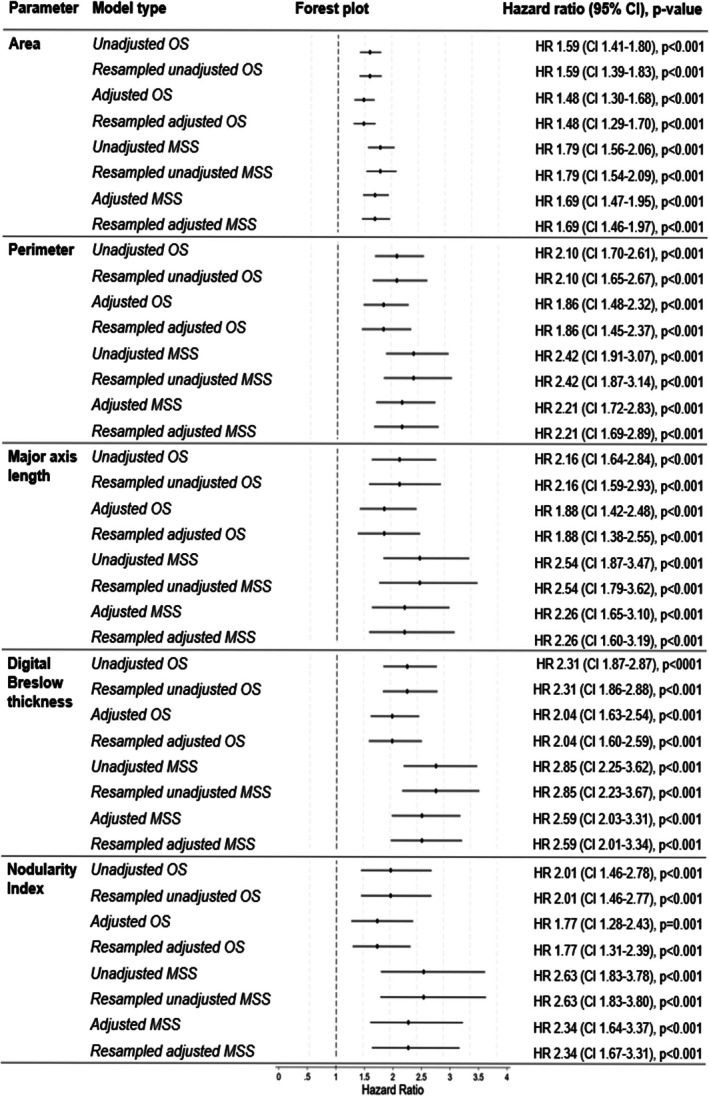
Hazard ratios and 95% confidence intervals (CI) for all log transformed parameters. Adjusted and unadjusted models for overall survival (OS) and melanoma specific survival (MSS) are presented, with adjusted models adjusted for age, sex and site.

Cox regression indicated that area *(log)*, perimeter *(log)*, major axis length *(log)*, dBT *(log)* and NI were all independently associated with overall survival (OS) and melanoma‐specific survival (MSS), after adjustment for age, sex and site. Resampling did not affect the results significantly. Hazard ratios and their 95% confidence intervals (CI) for each of the parameters can be found in Figure 3.

Digital Breslow thickness had a similar predictive power for OS and MSS, as compared with standard Breslow thickness [3]. The other parameters all had good predictive capabilities with concordance indexes of ≥0.67 (supplementary material, Table S2).

**Figure 4:**
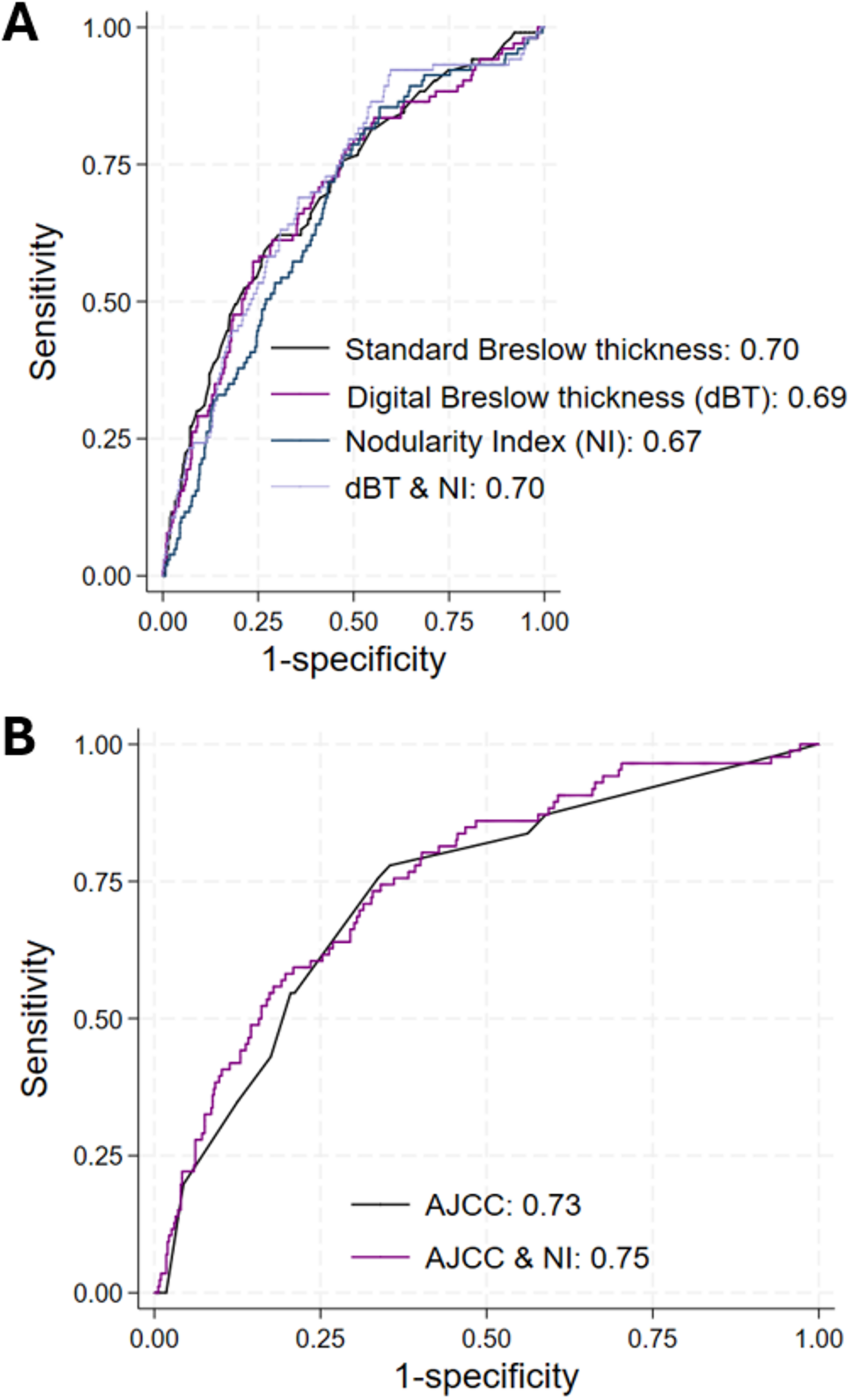
Receiver operating characteristic plots of melanoma‐specific survival at 5 years. (A) shows the area under the curve (AUC) for Breslow thickness, digital Breslow thickness, Nodularity Index, and their combination, whereas (B) shows the AUC for the clinical stage group (AJCC) and AJCC in conjunction with Nodularity Index. The dBT had a similar area under the curve (AUC) to Breslow thickness (0.69 *vs* 0.70). When dBT and NI were combined, the AUC increased to 0.70. AJCC alone achieved an AUC of 0.73, but when combined with NI, the AUC increased to 0.75, which represents a 3% improvement.

When comparing the models using logistic regression for melanoma‐specific survival at 5 years, dBT had a similar area under the curve (AUC) as standard Breslow Thickness (0.69 *vs*. 0.70). Moreover, when dBT and NI were combined, the AUC increased to 0.73. AJCC alone achieved an AUC of 0.74, but when combined with NI, the AUC increased to 0.76 (Figure 4), representing a 3% improvement.

### Subgroup analysis

There were statistically significant differences in tumour area, perimeter, major axis length, dBT and NI with ulceration status (present *vs*. absent), vascular invasion (present *vs*. absent), subtype (SSM *vs*. NM) and mitotic rate (≥1 per mm^2^ *vs*. <1 per mm^2^). Tumours with a higher area, a higher perimeter, a higher major axis length, a higher dBT and a higher NI were more likely to ulcerate. Tumours with vascular invasion were associated with a higher area, a higher perimeter, a higher major axis length, a higher dBT and a higher NI as compared with tumours without vascular invasion. Superficial spreading melanomas were associated with a lower area, a lower perimeter, a lower dBT and a lower NI, as compared with nodular melanomas. Tumours with a mitotic rate of ≥1 per mm^2^ were associated with a higher area, a higher perimeter, a higher major axis length, a higher dBT and a higher NI as compared with tumours with a lower mitotic rate.

There were statistically significant differences in tumour area, perimeter, major axis length and dBT with age (<50 years at time of diagnosis *vs*. ≥50 years). Tumour area, perimeter and major axis length varied with sex (male *vs*. female). Tumours from patients who were ≥50 years old at the time of diagnosis were associated with a higher area, a higher perimeter, a higher major axis length and a higher dBT. Tumours from male patients were associated with a higher area, a higher perimeter and a higher major axis length. However, there was no difference in NI by patient age or sex and there was no difference in dBT by sex.

There were no definitive differences in tumour size or shape in terms of site (central/peripheral), perineural invasion, regression, tumour infiltrating lymphocyte status (absent/non‐brisk/brisk), microsatellitosis or BRAF status. The average values for each of the tumour parameters for each subgroup can be found in supplementary material, Table S3.

## Discussion

This work demonstrates, as far as we are aware, the only instance of a neutral network being used to predict survival in melanoma using just tumour segmentation within a WSI. Many papers have utilized a multimodal approach, including clinicopathological information and/or gene expression profiles for survival prediction [9]. As according to a recent systematic review and meta‐analysis [12], those studies that have focused on the creation of melanoma segmentation models have assessed their use as a diagnostic aid or screening tool [13, 14, 15, 16, 17]. By contrast, the simplicity of our approach was taken to evaluate whether a bespoke CNN for invasive melanoma segmentation could be used to derive novel prognostic information without involving a histopathologist, rather than utilise existing data for survival prediction.

We have found that the ratio between the major and minor axis lengths derived by the CNN (minor axis length/major axis length)—the Nodularity Index (NI*log*)—independently predicts overall survival (HR 1.77 CI 1.28–2.43, *p* < 0.001) and melanoma‐specific survival (HR 2.34 CI 1.64–3.37, *p* < 0.001), with good predictive accuracy (Harrell's *C* statistic 0.68). The higher the NI, irrespective of size, the worse the outcome. Of note, the addition of NI to AJCC results is associated more closely with melanoma‐specific survival at 5 years, as compared with AJCC alone (AUC 0.73 *vs*. 0.75, respectively), which represents a 3% improvement on the current staging system. It is likely that the NI speaks to the underlying genetic landscape of the tumour—tumours with a higher NI are likely to bear mutations resulting in a more aggressive phenotype. We envisage that with time and further advancements in the objective morphological assessment of melanoma, the addition of multiple new parameters derived from neural networks will have a large cumulative effect on predictive accuracy.

We have also found that the CNN can be used to derive an objective ‘digital Breslow thickness’ (dBT), due to high correlation with standard Breslow thickness measurement (spearman's rho = 0.89). Similarly to its subjective predecessor, the digital Breslow thickness *(log)* is a strong independent predictor of overall (HR 2.04, CI 1.63–2.64, *p* < 0.001) and melanoma‐specific survival (HR 2.59, CI 2.03–3.31, *p* < 0.001), possesses the same discriminatory power (Harrell's *C* statistic 0.71 *vs*. 0.71 respectively) and confers a similar area under the curve for melanoma‐specific survival at 5 years (AUC 0.69 *vs*. 0.70).

A subgroup analysis evaluating CNN‐derived tumour size are reassuringly in accordance with our previous understanding of melanomas [18]—tumours that are larger are more likely to be ulcerated, be assigned a nodular subtype, come from an older or male patients and are more mitotically active.

A significant strength of this work is the robust method of evaluating the CNN's accuracy, as detailed in our previous paper [1]. The assessment involved multiple methodologies, including the use of 5 independent test sets for the per‐pixel evaluation of the CNN, before comparing the CNN's results to manual annotation of the whole tumour. This work also benefits from access to five data sources (2 internal, 3 external) with detailed accompanying clinical/genomic data and survival outcomes.

However, there are some notable limitations with this work. Despite 5 sources of data being used the requirement to contain only one WSI per patient containing conventional melanoma of full‐face, which had not been included in the training or validation sets, reduced the number of cases available to 745. Moreover, the limited size of the external test sets precluded separate analysis. Second, this data was all retrospectively collated and is therefore not yet tested on current tumours. Third, this study only assessed the CNN's application to the four major melanoma subtypes and did not include rarer tumour types, limiting the applicability. Finally, assessing the predictive accuracy of models is statistically challenging. Whilst the Harrell's *C* statistic is a useful tool for assessing relative predictive accuracies of models, it has limitations. For instance, it does not take into consideration the differences between the predicted probabilities and the observed outcomes and instead only assesses the ranked order of survival times. Therefore, it is important to be cautious in the interpretation of this statistic alone, and especially when the estimates are similar.

Future work will focus on the use of flexible parametric models to better understand the impact of the CNN‐derived parameters on survival, with adjusted hazard ratios per incremental change. Further survival modelling on further separate external retrospective data is underway to ensure robust generalizability before conducting a prospective trial. Finally, the melanoma area determined by the CNN will be compared with the CTA to determine if it may be regarded as a ‘digital CTA’.

To conclude, the custom‐designed CNN that segments invasive melanoma outlined in our previous work [1] has been used to create 5 histomorphological parameters of tumour size and shape, which appear to be associated with patient outcome: tumour area, tumour perimeter, major axis length, digital Breslow thickness (dBT) and Nodularity Index (NI). Given that these morphological biomarkers are automatically generated through segmentation, this model would provide an objective assessment of patient survival without impacting on clinical workflow.

In this initial work, the accuracy of the dBT provides reassurance in the results of the model, whilst the novel parameters, including tumour perimeter and NI, allude to the potential of this technology to become a rich source of new histological insights. Further work will be undertaken to validate these findings.

## Author contributions statement

EC: study design, funding acquisition, data curation, form–analysis, interpretation, writing (original draft). DM: analysis, interpretation, writing (review and editing), supervision. JNB: study design, funding acquisition, analysis, interpretation, writing (review and editing), supervision. GS: analysis, interpretation, writing (review and editing). WM: study design, interpretation, writing (review and editing). MH: formal analysis, interpretation, writing (review and editing). RI: study design, interpretation, writing (review and editing). NGM: analysis, interpretation, writing (review and editing). RAS: analysis, interpretation, writing (review and editing). GF: analysis, writing (review and editing). AA: analysis, writing (review and editing). MB: analysis, interpretation, writing (review and editing). ES: analysis, writing (review and editing). PK: analysis, writing (review and editing). SOS: study design, interpretation, writing (review and editing). DT: study design, funding acquisition, analysis, interpretation, writing (review and editing), supervision.

## Ethical approval statement

This work was conducted under the national system for ethical approval by the Cambridge East Research Ethics Committee (19/EE/0385).

## Supporting information


**Figure S1.** Major and minor axis lengths.
**Table S1.** Average values and their distribution for each of the tumour parameters for the largest piece of segmented tumour.
**Table S2.** Harrell's *C* statistic (concordance index) for each adjusted and log transformed parameter.
**Table S3.** Average values for each of the parameters by subgroup.

## Data Availability

The data that support the findings of this study are available from the University of Leeds, Leeds Teaching Hospitals NHS Trust, Melanoma Institute Australia, University of Leicester and Charles University and Teaching Hospital Kralovske Vinohrady, but restrictions apply and the data are not publicly available. Anonymized data are however available from the authors upon reasonable request and with permission from the relevant institution(s).
