# Supplementary material for: AI‐derived prognostic biomarkers from melanoma whole slide image segmentation: an initial discovery and assessment
Source: J Pathol Clin Res. 2026 Mar 4;12(2):e70075. doi: 10.1002/2056-4538.70075 (PMC12959248; doi:10.1002/2056-4538.70075)
Supplement: Supplementary file 1 — Figure S1. Major and minor axis lengths. Table S1. Average values and their distribution for each of the tumour parameters for the largest piece of segmented tumour. Table S2. Harrell's C statistic (concordance index) for each adjusted and log transformed parameter. Table S3. Average values for each of the parameters by subgroup. [file CJP2-12-e70075-s001.pdf]

AI-derived prognostic biomarkers from melanoma whole slide image segmentation: an initial discovery and assessment

Emily L Clarke et al J Pathol Clin Res https://doi.org/10.1002/2056-4538.70075

Supplementary Figure S1

Supplementary Tables S1 – S3

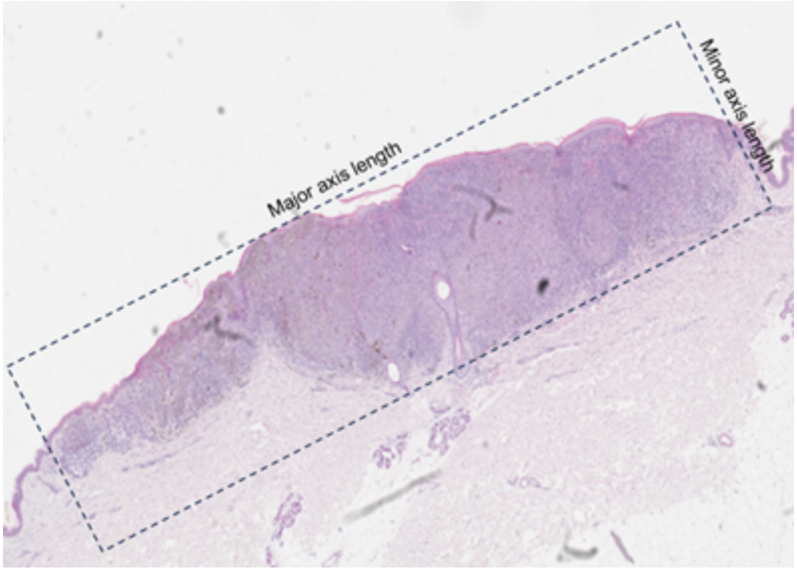

Figure S1. Major and minor axis lengths. Major and minor axis lengths are calculated using the rotating calipers algorithm to derive a bounding box encapsulating the tumour. The major axis length is the largest length of the bounding box and the minor axis length is the smallest. The Nodularity Index is a ratio between the major and minor axis lengths (minor axis length / major axis length) and is therefore a measure of shape independent of size.

Table S1. Average values and their distribution for each of the tumour parameters for the largest piece of segmented tumour.

	Median	IQR	Range
Area (mm^2)	6·1	3·3-13·8	0·1-275·1
Perimeter (mm)	18·3	12·2-27·6	1·5-122·0
Major axis length (mm)	5·8	4·2-8·1	0·5-25·6
Minor axis length (mm)	1·9	1·3-3·1	0·3-17·2
Nodularity Index	0·4	0·3-0·5	0·1-1·0

Table S2. Harrell's C statistic (concordance index) for each adjusted and log transformed parameter. All parameters were strong predicters with a concordance index of ≥0·67. The digital Breslow thickness had a similar concordance index as standard Breslow Thickness for both overall and melanoma specific survival.

CNN-derived parameter	Overall survival prediction Harrell's C statistic (concordance index)	Melanoma specific survival prediction Harrell's C statistic (concordance index)
Breslow Thickness	0·71	0·72
Digital Breslow thickness	0·71	0·71
Area	0·70	0·71
Perimeter	0·69	0·69
Major axis length	0·69	0·68
Nodularity Index	0·68	0·67

Table S3. Average values for each of the parameters by subgroup. Major axis length, digital Breslow thickness and Nodularity Index are derived for the largest piece of continuous tumour only· Data with a '*' are statistically significant (p<0·001) when compared to the other category·

Subgroup		Tumour area (mm^2)	Tumour perimeter (mm)	Major axis length (mm)	Digital Breslow thickness (mm)	Nodularity Index
Sex	Male	7·17*	19·99*	6·36*	2·03	0·36
	Female	5·24*	16·59*	5·45*	1·81	0·35
Age (years)	<50	5·21*	14·68*	5·02*	1·77*	0·35
	≥50	6·78*	19·49*	6·24*	1·99*	0·36
Site	Central	6·77	19·57	5·93	2·02	0·37
	Peripheral	5·57	17·10	5·71	1·81	0·34
Vascular invasion	No	5·42*	17·07*	5·55*	1·81*	0·35*
	Yes	12·28*	23·59*	7·17*	2·57*	0·41*
Perineural invasion	No	6·06	17·60	5·87	1·89	0·35
	Yes	6·43	18·61	5·80	1·99	0·36
Ulceration	No	4·42*	15·15*	5·11*	1·57*	0·33*
	Yes	13·56*	26·33*	7·61*	2·91*	0·40*
Regression	No	6·15	17·66	5·91	1·95	0·36
	Yes	5·73	17·93	5·38	1·83	0·34
Microsatellitosis	No	6·00*	18·13	5·81	1·90	0·35
	Yes	12·86*	26·99	8·29	2·59	0·39

Absent TILs	No	6·21	18·03	5·86	1·93	0·36
	Yes	10·32	21·15	6·62	2·40	0·40
Brisk TILs	No	6·73	18·21	5·86	2·00	0·37
	Yes	5·95	19·41	6·26	1·86	0·32
Subtype	SSM	4·67*	16·59*	5·48*	1·55*	0·31*
	NM	11·44*	21·95*	6·66*	2·58*	0·43*
Mitotic Rate	Low (<1 / mm^2)	3·85*	14·95*	4·89*	1·41*	0·31*
	High (≥1 / mm^2)	9·30*	21·32*	6·54*	2·39*	0·38*
BRAF status	Wildtype	5·55	18·95	5·78	1·99	0·37
	Mutant	6·55	19·39	5·83	1·87	0·33
